# Alloimmunization among transfusion-dependent thalassemia patients

**DOI:** 10.4103/0973-6247.53884

**Published:** 2009-07

**Authors:** Mohammad Hadi Sadeghian, Mohammad Reza Keramati, Zahra Badiei, Mehrangiz Ravarian, Hossein Ayatollahi, Houshang Rafatpanah, Mohammad Khajeh Daluei

**Affiliations:** 1 Department of Hematology and Blood Banking, Mashhad University of Medical Sciences, Mashhad, Iran; 2 Department of Pediatric, Mashhad University of Medical Sciences, Mashhad, Iran; 3 Department Immunology, Mashhad University of Medical Sciences, Mashhad, Iran; 4 Social Medicine, Mashhad University of Medical Sciences, Mashhad, Iran

**Keywords:** Alloantibody, thalassemia, transfusion

## Abstract

**Background:**

Thalassemia is a common hemoglobin disorder in Iran and one of the major public health problems. Although blood transfusions are lifesavers for thalassemia patients, they may be associated with some complications especially erythrocyte alloimmunization. The purpose of this study was to investigate the prevalence of red blood cell alloantibodies and to determine types of these antibodies among multiple-transfused thalassemic patients.

**Materials and Methods:**

A total of 313 thalassemia patients in the northeast of Iran, who received regular blood transfusion, were included in this study. Screening of antibodies was performed on fresh serum of all patients and then antibodies were identified in patients’ serum that had positive antibody screening test using a panel of recognized blood group antigens.

**Results:**

We identified 12 alloantibodies in 9 patients (2.87%) that all were against Rhesus (Rh) blood group antigens (D, C, E). Three patients developed 2 antibodies, and others had one antibody. The most common alloantibodies were Anti-D (88.88%) and followed by Anti-C and Anti-E. Higher frequency of alloimmunization was observed in female, Rh negative and splenectomized patients.

**Conclusion:**

This study showed that evaluation of the packed cells for Rh (C, E) from the start of transfusion can be helpful in decreasing the rate of alloantibody synthesis.

## Introduction

The thalassemia syndromes are a heterogeneous group of inherited disorders caused by genetic lesions leading to decreased synthesis of one or more of the globin subunits.[1] The globin chains that are produced in relative excess can damage the red cells or their precursors. As a result, there is an overall deficit of hemoglobin tetramers in the red blood cells (RBC) and the mean corpuscular volume (MCV) and mean corpuscular hemoglobin (MCH) are reduced.[2] Thalassemia is considered the most common genetic disorder worldwide. It occurs with a particularly high frequency in a broad belt extending from the Mediterranean basin through the Middle East (Iran), India and Southeast Asia. According to data collected from the Hereditary Disease Program of the World Health Organization (WHO) and based on local surveys and reports by visiting experts, the carriers of hemoglobin disorders in the world are estimated to be 269 million.[3] The only countries where a thalassemia register is maintained for surveillance purposes are Iran and Oman.[4,5]

Symptoms of the disease usually appear in the first year of life, as fetal hemoglobin (HbF) synthesis diminishes. Pallor is usually the first sign, accompanied by splenomegaly of various severity, fever, and failure to thrive. Affected children fail to develop and grow normally. Conventional treatment of beta thalassemia major is based on regular blood transfusion from early childhood, which improves the anemia and reduces the skeletal deformities associated with excessive erythropoiesis.[6,7] Although blood transfusion is a lifesaver for thalassemia patients, it may be associated with some complications such as iron overload, platelet and RBC alloimminization.[8‐10] Repetition of transfusions for the treatment of thalassemia major provokes the patient’s immune system and produces anti-erythrocyte antibodies (alloantibodies and/or autoantibodies). Erythrocyte autoantibodies appear less frequently, but they can result in clinical hemolysis and in difficulty in cross-matching blood. Alloimmunization against red blood cell antigens increases the need for transfusion and can be significantly complicated transfusion therapy. Some alloantibodies are hemolytic and may cause hemolytic transfusion reactions and limit the availability of further safe transfusion. Others are clinically insignificant.[11,12]

Ameen *et al*. reported 30% of thalassemia patients in Kuwait (southwestern neighbor of Iran) developed RBC alloantibodies. Although in southern Iran, alloantibodies were observed only among 5.3% of patients,[13,14] we could not find any documents about alloantibody frequencies in thalassemia patients in northeast Iran (Khorasan province).

Thalassemia is a common hemoglobin disorder in Iran and one of the major public health problems. However, limited data are available on the frequency of RBC alloimmunization in transfusion-dependent Iranian thalassemia patients. The purpose of this study was to determine the prevalence of RBC alloantibodies and the types of these antibodies among multiple-transfused thalassemic patients.

## Materials and Methods

This cross-sectional study was performed in Hemophilia and Thalassamia Center (Sarvar Clinic) in Mashhad, Iran between October 2006 and February 2008. Sarvar Clinic is the only referral center for diagnosis and treatment of hemophilia and thalassemia patients in the northeast of Iran. Most thalassemia patients from this clinic who received regular blood transfusion were included in this study. Transfusion strategy for compatibility matching was only for Rhesus D and ABO blood group in this center. The clinical findings of patients were recorded and the serological results were analyzed prospectively.

The patient’s age, sex, ABO and Rh blood group, symptoms, history of splenectomy, age of first blood transfusion, and years of blood transfusion were recorded. Also five milliliters of venous blood without anticoagulant was taken from each patient. After centrifugation, serum was separated and screened by standard blood bank procedures for detection of antibodies which uses three phenotypically recognized RBC samples at 37°C with proportion of two parts serum and one part RBCs. If agglutination did not occur after incubating for 60 minutes, polyclonal anti-human globulin (AHG) serum was added and negative / positive results were collected by light microscope. Finally alloantibodies were identified in sera of patients with positive antibody screening test using a panel of recognized blood group antigens [Table 1].

**Table 1:** Panel of red blood cells with recognized blood groups antigen

RBC	Donor No.	Rh						KELL				DUFFY		LUTHRAN		KIDD		MNS				P	Xga
		D	C	E	c	e	cw	K	k	Kpa	Kpb	Fya	Fyb	Lua	Lub	Jka	Jkb	M	N	S	s	P1	Xga
1	125376	+	−	−	+	+	−	+	+	−	+	+	−	+	+	+	+	+	−	−	+	−	−
2	496712	+	−	+	+	−	−	−	+	−	+	+	+	−	+	+	+	+	+	+	+	+	+
3	176414	+	+	+	+	+	−	−	+	−	+	+	−	−	+	+	+	+	−	−	+	+	+
4	0044650	+	+	−	−	+	−	−	+	−	+	−	+	+	+	+	+	−	+	−	+	+	+
5	004641	−	+	−	+	+	−	−	+	+	+	+	−	−	+	−	+	+	+	−	+	−	−
6	147130	−	−	+	+	+	−	−	+	−	+	+	−	−	+	+	+	+	+	+	+	+	+
7	140171	−	−	−	+	+	−	+	+	−	+	−	+	−	+	−	+	−	+	+	+	+	+
8	000080	−	+	−	+	+	−	−	+	−	+	+	+	−	+	+	−	+	+	−	+	+	+

Results from patients without alloantibody (group 1) and with alloantibody (group 2) were analyzed with SPSS (Statistical Software for Social analysis-version 11.5) by a statistician and using T test, Chi-Square, and Fisher’s Exact Test. A P-value less than 0.05 was considered significant.

## Results

Clinical and laboratory data from 313 thalassemia patients, 187 (59.7%) male and 126 (40.3%) female were analyzed. The age range was from 8 month to 38 years with 14.42 ± 7.59 for mean ± standard deviation (SD). Four cases (1.2%) received blood transfusions at 2 weeks interval (390cc/kg/year); 219 cases (70%) at 3 weeks interval (260 cc/kg/year); 36 cases (11.4%) at 4 weeks interval (195 cc/kg/year); 54 cases (17.4%) at more than 4 weeks interval or with irregular blood transfusion. Twelve alloantibodies were found in 9 (2.87%) patients (6 females and 3 males). Three patients detected two antibodies, and others had only one antibody. The most common alloantibody was Anti-D (88.88%) followed by Anti-C (33.33%) and Anti-E (11.11%). Three patients (33.33%) had two different alloantibodies. Higher frequency of alloimmunization was detected in females (male: female = 1:2), Rh negative blood group, and splenectomized patients [Table 2].

**Table 2:** Laboratory and clinical finding in the thalassemia patients with alloantibody

Number	Antibody	Sex (years)	Age	Splenectomy transfusion (years)	Duration of Blood group	ABO	Rh
1	Anti-D and Anti-C	female	20.00	yes	16.00	A	−
2	Anti-D	female	19.00	yes	18.50	B	−
3	Anti-D	male	23.00	yes	22.50	A	−
4	Anti-D	female	11.00	yes	10.50	A	−
5	Anti-D and Anti-C	female	9.00	no	5.00	A	−
6	Anti-D	male	9.00	yes	8.50	O	−
7	Anti-D and Anti-C	female	24.00	yes	22	B	−
8	Anti-E	male	9.00	yes	8	A	+
9	Anti-D	female	10.00	no	9	A	−

Table 3 shows the distribution of ABO and Rh blood group in thalassemic patients. Chi-Square test showed significant differences (Odd ratio = 0.012) for Rh blood group between patients without alloantibody (group 1) and patients with alloantibody (group 2). Mean age for group 1 and 2 was 14.04 and 14.88 years, respectively, which was not significant (P = 0.85). Male to female ratio was 1.5/1 and ½ in subject of groups 1 and 2, respectively. The gender differences were not significant between the two groups (P = 0.09). Seventy-seven point eight vs. 32.2% of patients with and without alloantibody had undergone splenectomy, respectively. Fisher’s Exact Test showed significant differences between splenectomy and alloimunization (P = 0.008).

**Table 3:** Frequency of ABO and Rh blood group in thalassemic patients

	A	B	AB	O	Rh	

					Positive	negative
Group 1*	32.4%	23.8%	7%	36.8%	88.5%	11.5%
Group 1**	66.7%	22.2%	0%	11.1%	11.1%	88.9%

* Patients without alloantibody

** Patients with alloantibody

The mean duration of transfusion (the years between first patient’s transfusion and the time of our study) was 10.5 and 13.2 years in groups 1 and 2, respectively, and T test showed no significant differences (*P* = 0.25). Furthermore, no significant differences were observed between the two groups for total blood infusion during the time period (*P* = 0.20). The percent of patients that had received washed red blood cells and packed RBC with a leukoreduced filter for group 1 was 16.8 and 83.2%, respectively, and for group 2 was 22.2 and 77.8%, respectively. There were no significant differences between the two (*P* = 0.88).

## Discussion

Thalassemia was first reported in the literature in 1925, when Cooley and Lee described a form of severe anemia, occurring in children and associated with bone changes and splenomegaly. Although bone marrow transplantation is the only cure, regular blood transfusion is available treatment for these patients.[15] Early and regular blood transfusion therapy in patients with thalassemia decreases the complications of severe anemia and prolongs survival. In the long term, however, the beneficial effects of transfusions are limited by complications such as chronic viral infections, hemosiderosis and alloimmunization against RBC.[6]

Our results indicated that the frequency of alloimunization in thalassemia patients in northeast Iran is 2.87%. This frequency has been reported in 30% of 190 thalassemia patients in Kuwait, 4.97% of 161 in Indian patients, 5% of 1435 Italian patients and also 3.7% of 1200 thalassemia patients in Greece.[13,10,16,17] There are also a few similar studies in Iran. In one study that was performed on 711 thalassemia patients in Shiraz (in southern Iran) 38 (5%) patients had red cell antibodies.[14] The prevalence of alloimunization in our study was low compared with the above studies. This may be due to selection of thalassemia patients who all had the severe form of disease (major thalassemia) or intermediate form. Furthermore selection of patients with low age and low transfusion rate in our study may contribute to alloimunization prevalence. Frequency of alloimunization was 4.5% if we excluded the results of patients with low transfusion rate. Also the prevalence of thalassemia is low in northeast of Iran. Thalassemia is more prevalent in the northern (Caspian Sea coast) and southern (Persian Gulf and Oman Sea coasts) areas of Iran so the geographic characteristics can be implicated for mismatch prevalence results.[18]

All of our patients’ alloantibodies were against the Rh system (Anti-D, Anti-C and Anti-E). The Rh system antibodies are important in transfusion medicine because these antibodies can cause hemolytic transfusion reactions.[19] In a Bhatti *et al*. study, RBC alloantibodies belonged mainly to Rh system although other antibodies such as anti-K, anti-Jsb and anti-Jka were detected.[10] Furthermore the most common clinically significant alloantibodies that were detected in an Ameen *et al*. study were directed against antigens in the Kell and Rh systems. Anti-K in 41 (72%) patients and anti-E in 26 (45.6%) were reported.[13] In another study, Sirchia *et al*. collected clinical and laboratory data on Italian thalassemia major patients and detected 136 alloantibodies in 74 thalassemia patients that were against the antigens of the Rh, Kell, Kidd, and Duffy systems.[16]

Karimi *et al*. reported high prevalence of antibodies (47.7%) that were against the Rh system.[14] Although high prevalence of Anti-Rh was reported in previous studies, the frequency of Anti-D (88.88%) in our study was very common. One of the most important reasons for this alloimmunization was transfusion of some red blood cells with rhesus D incompatible with thalassemia patients due to false negative results in weak D typing of blood donors. Transfusions of weak D (D positive) red blood cells to D negative patients stimulate the immune system for production of anti-D. Approximately 1% of D-positive individuals type as weak D (historically known as D^U^), characterized by weak or absent RBC agglutination by anti-D during routine serologic testing. In weak D individuals, the D antigen usually requires enhancement with antihuman globulin (AHG). Weak D typing is routinely done in all blood donors in Iran. Repeat testing of donor units by the transfusion service for weak D is not required by the American Association of Blood Banks (AABB) Standards and is not performed in Iranian transfusion services, but these centers reconfirm the D antigen of all RBC units labeled Rh-negative by testing red cells from an integral attached segment by a direct anti-D agglutination method. We did not find any records of the exact prevalence of weak D in Iran but prevalence of Rh D negative was reported as 10.2% by Ayatollahi *et al*. in subjects of northeast Iran.[20] It is obvious that more attention to quality control programs in blood banking laboratory is necessary especially for determination of weak D positive RBCs and reporting of D negative blood group. Furthermore antibody screening should be performed for Rh (D, C and E) antigens as the most common alloantibodies in all of the patients from the start of transfusion to reduce transfusion complications.

## Conclusion

High frequency of Anti-D in our study implies that more attention should be paid to quality control programs for determination of weak D positive red blood cells, and in order to decrease the rate of alloantibody synthesis, transfusion of matched packed RBCs for minor blood groups, especially for Rh (C, E) should be considered in addition to major blood groups (ABO, Rh-D) from the start of transfusion.
